# Early neurological rehabilitation management in a stroke patient with persistently and extremely elevated natriuretic peptides: a case report

**DOI:** 10.3389/fmed.2026.1809255

**Published:** 2026-03-19

**Authors:** Xinyuan Han, Zhijun Huang

**Affiliations:** Department of Neurological Rehabilitation, Shaanxi Provincial Rehabilitation Hospital, Xi’an, China

**Keywords:** cardiovascular reserve, case report, multidisciplinary management, natriuretic peptides, neurological rehabilitation, stroke

## Abstract

**Background:**

A marked elevation in N-terminal pro-B-type natriuretic peptide (NT-proBNP) typically suggests heart failure. However, its clinical significance in acute stroke patients without heart failure symptoms remains unclear, often complicating decisions regarding early rehabilitation. When combined with comorbidities such as renal insufficiency, the marked contrast between extremely high NT-proBNP levels and a stable clinical presentation poses a significant challenge for safely initiating rehabilitation.

**Case description:**

This report describes a 65-year-old female admitted with an acute left basal ganglia and periventricular cerebral infarction. She had comorbid diabetic kidney disease (stage IV) and renal anemia. Following the stroke, her NT-proBNP level remained persistently elevated above 20,000 pg/mL, yet she was clinically stable with normal cardiac structure and function on resting echocardiography. We interpreted this condition as a state of “cardiovascular reserve exhaustion under multiple hits” and subsequently formulated an individualized rehabilitation strategy based on “cautious initiation and stepwise progression.” Through multidisciplinary collaboration, we optimized her volume status and corrected anemia, implementing a stepwise rehabilitation protocol under rigorous monitoring. The patient successfully completed rehabilitation without cardiovascular adverse events and demonstrated neurological improvement.

**Conclusion:**

In stroke patients with complex comorbidities, persistently and markedly elevated NT-proBNP levels may reflect a state of “cardiovascular reserve exhaustion” rather than simple heart failure. Under the premise of rigorous multidisciplinary management, optimization of reversible factors, and close monitoring, individualized stepwise early rehabilitation may not necessarily require delay due to this isolated abnormality. This approach suggests that neurological recovery can be promoted while ensuring safety.

## Introduction

Elevated levels of NT-proBNP following stroke often complicate clinical management (1, 2). Although this biomarker is strongly associated with heart failure, its pathophysiological significance and clinical implications in stroke patients without overt heart failure symptoms remain unclear (3), frequently creating a dilemma in the decision-making process for initiating early neurological rehabilitation. This challenge is particularly pronounced in patients with complex comorbidities such as severe renal insufficiency. A marked discrepancy between extremely high natriuretic peptide levels and a relatively stable clinical presentation turns the selection of rehabilitation timing and intensity into a clinical conundrum requiring multidisciplinary collaboration (4). Currently, there is a lack of management consensus for such complex cases. An overly conservative approach may delay crucial rehabilitation, while an imprudent, aggressive one could precipitate cardiovascular events.

This case report aims to address this gap by presenting a typical case to dissect the complex mechanisms underlying significantly elevated natriuretic peptides post-stroke. We propose an integrated management strategy centered on the core concept of “cardiovascular reserve exhaustion.” This concept posits that the cumulative burden of chronic comorbidities (e.g., hypertension, chronic kidney disease, anemia) establishes a baseline of compromised cardiac reserve; an acute insult (such as stroke-induced sympathetic storm) further depletes this reserve; and impaired renal clearance amplifies biomarker signals. It should be emphasized that this concept is a hypothesis-generating construct based on pathophysiological reasoning, intended to provide an integrative thinking framework for rehabilitation decision-making in patients with complex comorbidities. Its validity requires further research and validation. We detail how, guided by this framework, a multidisciplinary assessment and rigorous monitoring protocol were employed to implement an individualized, stepwise early rehabilitation plan. This approach sought to balance neurological recovery with cardiovascular safety, thereby providing empirical evidence and a conceptual reference for managing this common clinical dilemma.

## Case description

**Figure 1:**
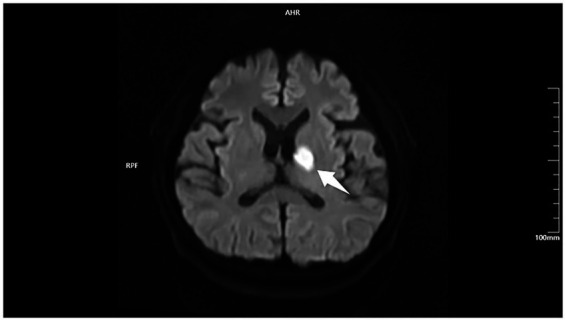
Admission brain diffusion-weighted imaging (DWI). Axial image shows an acute infarct in the left basal ganglia and periventricular region, presenting as marked hyperintensity (arrow). This lesion correlates with the patient’s right-sided hemiparesis, consistent with the imaging diagnosis of acute ischemic stroke.

A 65-year-old female was admitted due to “sudden-onset right-sided limb weakness for 12 hours.” Her past medical history included a 15-year history of hypertension with fair control, and a 12-year history of type 2 diabetes mellitus which had progressed to diabetic kidney disease, stage IV. She also had chronic renal anemia. On admission, her vital signs were stable: temperature 36.4 °C, heart rate 76 beats per minute, respiratory rate 20 breaths per minute, and blood pressure 138/82 mmHg. There were no signs of dyspnea or volume overload. Neurological examination revealed right upper limb muscle strength was grade 1, and right lower limb muscle strength was grade 2 (using manual muscle testing, MMT). Brain magnetic resonance imaging (MRI) confirmed an acute left basal ganglia and periventricular infarction (Figure 1).

Guideline-based acute stroke therapy was initiated immediately upon admission, consisting of aspirin 100 mg daily, clopidogrel 75 mg daily, and atorvastatin 20 mg daily. Laboratory investigations showed: NT-proBNP 23,100 pg/mL; serum creatinine 256 μmol/L (estimated glomerular filtration rate [eGFR] 22 mL/min/1.73 m^2^); hemoglobin 75 g/L. Cardiac evaluation revealed T-wave inversion in leads V2–V5 on electrocardiogram (ECG). Transthoracic echocardiography (TTE) demonstrated preserved left ventricular ejection fraction (LVEF) of 56%, no structural abnormalities, and normal diastolic function (*E*/*e*′ ratio of 8).

Based on the clinical reasoning of “cardiovascular reserve exhaustion under multiple hits,” the treatment goal was defined as safely advancing neurological rehabilitation while prioritizing the stabilization of cardiac and renal status. Within 72 h of symptom onset, we implemented multidisciplinary integrated management in collaboration with the cardiology and nephrology departments, alongside standard neurological care. Volume management was guided by strict monitoring of daily fluid balance, body weight, and clinical presentation, with cautious use of diuretics. Two units of packed red blood cells were transfused to correct the hemoglobin level to 92 g/L. Blood pressure was maintained within a stable range of 130–140/80–85 mmHg. Following renal function assessment, a comprehensive cardio-renal protective medication regimen was initiated. These measures aimed to establish a stable physiological foundation for subsequent rehabilitation.

**Figure 2:**
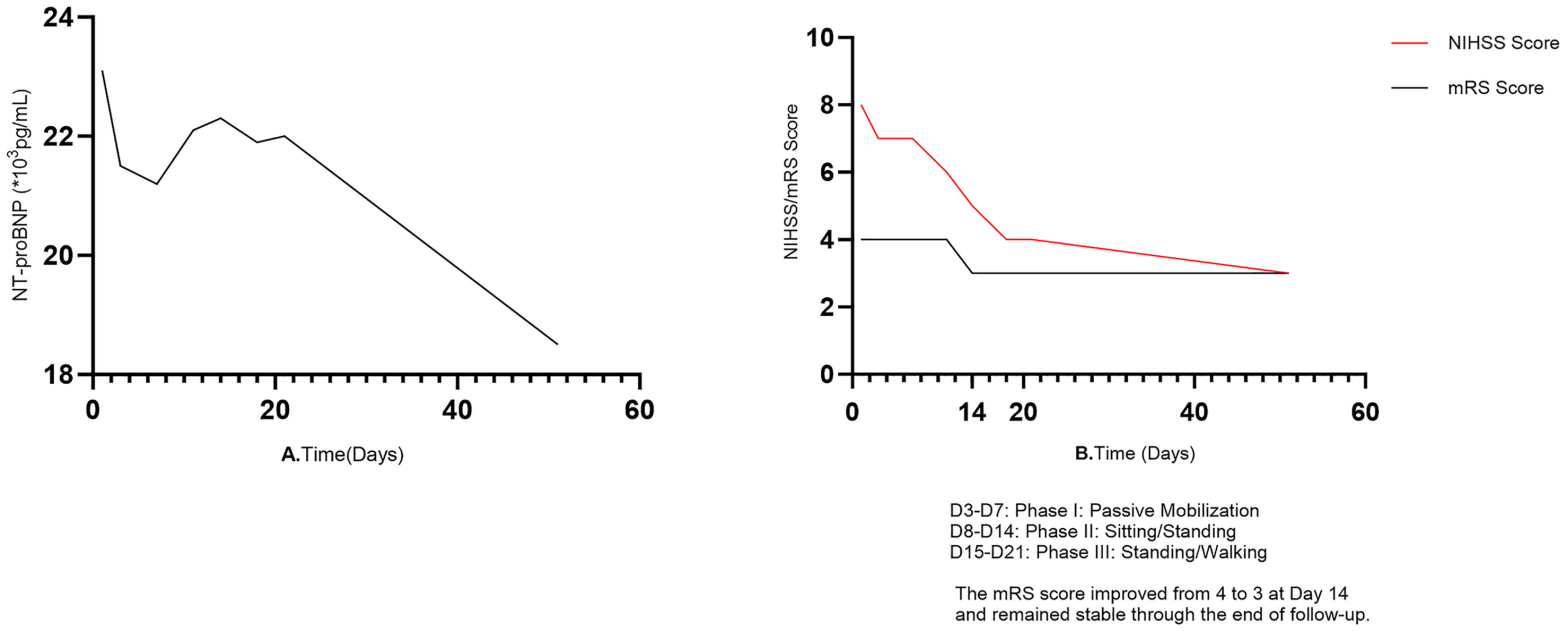
**(A)** Dynamic trend of NT-proBNP during the management period. The line corresponds to the left *Y*-axis, representing serum NT-proBNP levels (units: ×10^3^ pg/mL). On admission (Day 1), NT-proBNP was 23.1 × 10^3^ pg/mL. During the stepwise rehabilitation phases (Phase I: Days 3–7, passive mobilization; Phase II: Days 8–14, sitting/standing; Phase III: Days 15–21, standing/walking), NT-proBNP fluctuated within a high but stable range of 21–23 × 10^3^ pg/mL, declining to 18.5 × 10^3^ pg/mL at the one-month follow-up. **(B)** Trends in neurological function and disability level during the management period. The orange line (NIHSS score) and black line (mRS score) both correspond to the left *Y*-axis. The NIHSS score decreased from 8 on admission to 3 at follow-up, indicating neurological improvement. The mRS score improved from 4 to 3 on Day 14 and remained stable thereafter.

Under continuous ECG, blood pressure, and oxygen saturation monitoring, an individualized rehabilitation protocol based on a “cautious initiation and stepwise progression” strategy was commenced on day 3 post-stroke. Each rehabilitation session lasted 15–30 min and was conducted once or twice daily, with adjustments made based on patient tolerance. Heart rate and blood pressure were monitored during postural changes (e.g., from supine to sitting, and from sitting to standing); no symptomatic orthostatic hypotension occurred. During the first week, passive range-of-motion exercises were performed in bed. The patient tolerated this well, with NT-proBNP levels fluctuating between 21,000 and 23,000 pg/mL. In the second week, rehabilitation was escalated to include sitting at the bedside and assisted standing. The patient remained hemodynamically stable; right upper limb muscle strength improved to grade 2, and lower limb strength to grade 3. NT-proBNP levels remained around 22,000 pg/mL. During the third week, rehabilitation progressed further to include standing balance training and assisted walking within parallel bars. The patient was able to ambulate short distances; right upper limb strength reached grade 3, and lower limb strength grade 4. NT-proBNP levels did not show any further increase. Throughout the entire stepwise rehabilitation period, the patient underwent continuous ECG monitoring, symptom surveillance, and periodic re-evaluation of myocardial injury markers (troponin). She did not develop any signs or symptoms of heart failure, myocardial ischemia (such as angina or dynamic ST-T changes), or new-onset arrhythmias, indicating the absence of cardiovascular adverse events. After 3 weeks of treatment, the patient’s neurological function showed significant improvement: the National Institutes of Health Stroke Scale (NIHSS) score decreased from 8 to 4, and the modified Rankin scale (mRS) score improved from 4 to 3. A summary of the key treatment processes, dynamic changes in indicators, and outcomes from admission to 1 month post-discharge is presented in Table 1 and Figures 2A,B. At the one-month follow-up after discharge, the NT-proBNP level had gradually decreased to 18,500 pg/mL; the NIHSS score further improved to 3, and the mRS score remained at 3. Thereafter, telephone follow-up was continued for 3 months (twice monthly), during which the patient reported stable condition, no symptoms, and no readmission.

**Table 1:** Evolution of clinical management, rehabilitation phases, and key indicators.

Time point	Core treatment & rehabilitation phase	Clinical presentation & vital signs	NT-proBNP (pg/mL)	Troponin (ng/mL)	Hemoglobin (g/L)	Creatinine (μmol/L)	Blood pressure range (mmHg)	NIHSS score	mRS score
Admission (D1)	Diagnosis & initiation of acute-phase therapy	Right-sided hemiplegia, stable vital signs	23,100	<0.01	75	256	138/82	8	4
D3	Initiation of multidisciplinary integrated management	No discomfort	21,500	<0.01	75 → 92^a^	256	135/82	7	4
D7	Passive mobilization phase	Well tolerated, normal vital signs	21,200	<0.01	92	248	130–140/80–85	7	4
D11	Sitting/Standing training phase	No discomfort, normal heart rate response upon standing	22,100	<0.01	91	245	130–140/80–85	6	4
D14	Sitting/Standing training phase	Able to stand with assistance, stable vital signs	22,300	<0.01	90	242	130–140/80–85	5	3
D18	Walking training phase	Able to ambulate short distances, no shortness of breath	21,900	<0.01	89	238	130–140/80–85	4	3
D21 (discharge)	Inpatient rehabilitation conclusion	Able to walk slowly with assistance, stable condition	22,000	<0.01	88	235	130–140/80–85	4	3
1 month post-discharge	Outpatient rehabilitation follow-up	Condition stable, function continued to improve	18,500	—^b^	90	230	Stable	3	3

a Two units of packed red blood cells were transfused on D3, increasing hemoglobin from 75 g/L to 92 g/L.

b Troponin was not reassessed at the one-month follow-up. Reference ranges (institution-specific): troponin <0.04 ng/mL; hemoglobin 115–150 g/L; creatinine 40–80 μmol/L. Blood pressure ranges are summarized from daily monitoring. NT-proBNP values are typical measurements obtained near the respective time points (after appropriate rounding). NIHSS, National Institutes of Health Stroke Scale (a lower score indicates better neurological function); mRS, modified Rankin scale (score range 0–6, a lower score indicates better function).

## Discussion

This case illustrates an interdisciplinary dilemma in neurological practice: how to formulate a rehabilitation strategy for post-stroke patients with persistently and markedly elevated NT-proBNP levels but without clear evidence of heart failure. Initiating routine rehabilitation training risks inducing acute cardiac decompensation, yet withholding rehabilitation due to abnormal laboratory findings may lead to complications such as disuse syndrome and deep vein thrombosis, severely impacting neurological outcomes. Therefore, a balance between risk and benefit must be sought.

Traditionally, NT-proBNP is primarily used to aid in the diagnosis and severity assessment of heart failure (5). In this case, the patient exhibited no signs or symptoms of heart failure, and resting TTE showed normal cardiac structure and systolic function. The diastolic function parameter (*E*/*e*′ 8) could not adequately explain the extreme elevation of NT-proBNP. However, it must be acknowledged that resting *E*/*e*′ alone cannot completely exclude the possibility of heart failure with preserved ejection fraction (HFpEF). In this case, the absence of heart failure manifestations should be interpreted as no clinical decompensation was detected, rather than heart failure being completely excluded. Consequently, we need to move beyond the binary “heart failure present or absent” mindset. The underlying pathophysiology involves an integrated effect of multiple factors. The acute cerebral infarction likely triggered sympathetic overactivation and neuroendocrine dysregulation (i.e., the “brain-heart syndrome”), which can lead to myocardial stunning and natriuretic peptide release (6–8). Concurrently, severe renal insufficiency (eGFR 22 mL/min/1.73 m^2^) significantly reduced the clearance of NT-proBNP, leading to its accumulation in the blood (9). The T-wave inversions observed in ECG leads V2–V5, while potentially indicative of chronic coronary ischemic changes, more likely suggested diffuse myocardial repolarization abnormalities related to the brain-heart syndrome or multifactorial myocardial pathology in the absence of acute myocardial injury evidence (10–12). Therefore, the markedly elevated NT-proBNP level in this patient is best understood as the result of the combined effects of an acute cerebral ischemic insult and renal clearance impairment, superimposed on a baseline of chronic cardiac and renal disease (13). This finding should be interpreted as a strong signal for a “cardiovascular reserve exhaustion” state preceding symptomatic heart failure (14), rather than a mere marker of heart failure per se.

This pathophysiological understanding prompted a reframing of the neurological rehabilitation decision-making framework. The focus of risk consideration shifted from “whether heart failure will be induced” to “whether the individual’s cardiovascular reserve limit will be exceeded.” This shift necessitates a transition from a binary decision to a dynamic balancing act in management strategy. The core of our adopted strategy, “cautious initiation and stepwise progression,” lies in transforming the rehabilitation activities themselves into a series of meticulous “monitored progressive load assessment” (15). Each incremental increase in intensity was undertaken only after multidisciplinary optimization of foundational therapy (e.g., volume management, anemia correction, and initiation of cardio-renal protective medications) (16), and was coupled with continuous physiological monitoring to dynamically explore the patient’s safe tolerance window. In this case, NT-proBNP remained stable at a high level without further elevation as rehabilitation progressed. Combined with the absence of abnormalities on continuous ECG monitoring and troponin measurements, these findings suggest that the strategy was well tolerated physiologically within the monitored parameters, providing supportive evidence for its safety. Notably, the assessment of cardiac safety should employ multiparameter monitoring. In this case, in addition to NT-proBNP, clinical symptoms, ECG, and myocardial injury markers (troponin) were simultaneously monitored. All parameters remained stable throughout the rehabilitation period, collectively forming an evidence chain for safety evaluation. Given the patient’s background of diabetic kidney disease stage IV, the clinical significance of NT-proBNP should focus more on dynamic trends rather than a single measurement. As noted in JACC: Heart Failure, elevated NT-proBNP in heart failure and chronic kidney disease is a true warning signal of worsening prognosis, rather than a false positive (17). In this case, NT-proBNP remained stable at a high level throughout the rehabilitation period, further corroborating the safety of the rehabilitation protocol.

It is recommended that future prospective studies include regular follow-up of at least 6 months with objective parameters such as NT-proBNP, troponin, and echocardiography to comprehensively evaluate long-term cardiovascular outcomes. However, the neurological improvement observed in this case may partly reflect the natural recovery process following stroke. The single-case design cannot distinguish intervention effects from spontaneous recovery; therefore, this study aims only to demonstrate the feasibility of the rehabilitation protocol, rather than to prove its superiority.

## Conclusion

For stroke patients with complex cardio-renal comorbidities, persistently and extremely elevated NT-proBNP levels more likely signify “cardiovascular reserve exhaustion” rather than simple heart failure. However, it should be emphasized that this case only demonstrates the absence of detected clinical decompensation, rather than the complete exclusion of heart failure. By optimizing the patient’s baseline status through multidisciplinary collaboration and implementing stepwise rehabilitation as a tightly monitored progressive load assessment, neurological rehabilitation can be safely advanced, thereby improving functional outcomes (18).

## Limitations

This study is a single-case report. The proposed “cardiovascular reserve exhaustion” concept and management strategy stem from the pathophysiological reasoning and management experience of this specific case. Limited by available resources, this study did not employ imaging techniques such as cardiac magnetic resonance or stress echocardiography, nor did it measure objective stress indicators including oxygen consumption and lactate levels. Consequently, a comprehensive investigation of myocardial, microcirculatory, and cardiac reserve function was not achieved. These factors collectively limit the comprehensiveness of the assessment of the patient’s cardiac reserve function. In addition, the specific steps and intensities of the rehabilitation protocol were based on the clinical team’s judgment and have not yet been standardized into an operational protocol, nor were objective parameters such as metabolic equivalents (METs), heart rate reserve, or Borg scale ratings employed to quantify exercise intensity, or clear safety termination thresholds pre-specified. Nonetheless, the clinical problem highlighted, the integrated analytical approach, and the safety-oriented management principles demonstrated by this case retain significant instructive and referential value for clinical practice. It should be emphasized that the management strategy proposed in this study derives from a single-case experience and is not intended to replace current guideline-based cardiac evaluation protocols. Its safety and efficacy require further validation through prospective cohort studies or randomized controlled trials before broader clinical application. Furthermore, objective follow-up data were available only for the first month, with the subsequent 3 months relying on telephone interviews. The lack of laboratory and imaging assessments limits the precision of long-term outcome interpretation.

## Patient perspective

The patient and her family expressed that they initially felt considerable fear upon learning about the “extremely high cardiac marker,” worrying that rehabilitation would be impossible. After the healthcare team provided a detailed explanation of the “reserve” concept and the stepwise rehabilitation plan, they understood the rationale behind the treatment approach. They cooperated actively with the monitoring and treatment process. Ultimately, they expressed satisfaction with achieving functional recovery under conditions they perceived as safe.

## Data Availability

The original contributions presented in the study are included in the article/supplementary material, further inquiries can be directed to the corresponding author.
